# Fatigue and depressive symptoms improve but remain negatively related to work functioning over 18 months after return to work in cancer patients

**DOI:** 10.1007/s11764-018-0676-x

**Published:** 2018-02-05

**Authors:** H. F. Dorland, F. I. Abma, S. K. R. Van Zon, R. E. Stewart, B. C. Amick, A. V. Ranchor, C. A. M. Roelen, U. Bültmann

**Affiliations:** 1 Department of Health Sciences, Community and Occupational Medicine, University of Groningen, University Medical Center Groningen, Antonius Deusinglaan 1, FA10, Room 417, 9713 AV Groningen, The Netherlands; 2 Robert Stempel College of Public Health and Social Work, Department of Health Policy and Management, Florida International University, Miami, FL USA; 3 Institute for Work and Health, Toronto, Canada; 4 Department of Health Psychology, University of Groningen, University Medical Center Groningen, Groningen, the Netherlands; 5 HumanCapitalCare, Enschede, the Netherlands

**Keywords:** Cancer, Work functioning, Cognitive symptoms, Depressive symptoms, Workplace social support

## Abstract

**Purpose:**

The aims of this study are to investigate the course of work functioning, health status, and work-related factors among cancer patients during 18 months after return to work (RTW) and to examine the associations between these variables and work functioning over time.

**Methods:**

Data were used from the 18-month longitudinal “Work Life after Cancer” (WOLICA) cohort, among 384 cancer patients who resumed work. Linear mixed models were performed to examine the different courses during 18-month follow-up. Linear regression analyses with generalized estimating equations (GEE) were used to examine the associations and interactions.

**Results:**

Cancer patients reported an increase of work functioning and a decrease of fatigue and depressive symptoms in the first 12 months, followed by a stable course between 12 and 18 months. Cognitive symptoms were stable during the first 18 months. Working hours increased and social support decreased during the first 6 months; both remained stable between 6 and 18 months. Fatigue, depressive, and cognitive symptoms were negatively associated with work functioning over time; working hours and supervisor social support were positively associated.

**Conclusions:**

Interventions to improve cancer patients’ work functioning over time might be promising if they are aimed at reducing fatigue, depressive symptoms, cognitive symptoms, and encouraging supervisor social support.

**Implications for Cancer Survivors:**

It is important to monitor cancer patients not only in the period directly after RTW but up to 18 months after RTW, allowing for timely interventions when needed.

## Introduction

Survival rates of cancer patients are increasing, due to improvements in cancer diagnosis and treatment [1, 2]. More than 60% of cancer patients return to work (RTW) within 1–2 years after cancer diagnosis globally [3]. However, cancer patients can experience difficulties when returning to work due to cancer treatment or as a result of psychological symptoms related to cancer diagnosis [4, 5]. Health-related work functioning (hereafter referred to as work functioning) measures the ability to meet the demands of work for a given state of health [6–8]. Work functioning reflects the interplay between work and health and might therefore be seen as a highly valuable outcome [6–8].

Earlier, we have identified three distinct work functioning trajectories in the year following RTW of cancer patients, and baseline cognitive symptoms, time between diagnosis and RTW, and changed meaning of work were associated with these trajectories [9]. To date, knowledge about the course of work functioning in cancer patients during the first 18 months after RTW is lacking. Moreover, little is known about how health status and work-related factors (i.e., work demands and social support) change over time and information about their influence on work functioning over time in cancer patients is lacking. This knowledge is important for physicians who treat cancer patients with paid work and for the development of interventions to improve work functioning of cancer patients.

Therefore, the aims of this study are (1) to investigate the course of work functioning, health status, and work-related factors in cancer patients during 18 months after RTW and (2) to examine the associations between health status and work-related factors with work functioning over time.

## Material and methods

### Study design and sample

The Work Life after Cancer (WOLICA) study is an 18-month longitudinal cohort study among cancer patients. Cancer patients were included when they (1) were between 18 and 65 years old and (2) had resumed work for at least 12 h/week during or following primary cancer treatment. Exclusion criteria were (1) recurrent cancer, (2) treated with palliative intent, (3) no paid employment for at least 1 year prior to cancer diagnosis, and (4) not able to complete a questionnaire in Dutch. Potential participants were identified and asked by their Occupational Physician (OP) if they were interested to participate in this study. If interested, OPs forwarded the cancer patients’ name and address to the research team. Cancer patients who met the inclusion criteria received additional information about the study, an informed consent, and the baseline questionnaire. Cancer patients were included at baseline within the first 3 months of working ≥ 12 h/week. Participants received follow-up questionnaires, measuring socio-demographics, health status, and work-related factors every 3 months. WOLICA was reviewed and approved by the Medical Ethical Committee of the University Medical Center Groningen (M12.125242). A detailed description of WOLICA has previously been reported [9].

### Measures and procedure

#### Socio-demographics

Baseline age (in years), gender (male; female (ref)), education (low, i.e., primary, junior secondary vocational, and junior general secondary education; medium, i.e., senior secondary vocational education and senior general secondary education; high (ref), i.e., higher professional education, college, and university), and marital status (single/divorced; married/cohabitating (ref)) were used as covariates.

### Work functioning

Work functioning was measured at baseline, 6, 12, and 18 months after RTW with the Work Role Functioning Questionnaire (WRFQ 2.0; 27 items, α = 0.96) [8], a reliable and validated questionnaire designed to measure difficulties in meeting work demands perceived by workers with physical health problems or emotional problems in the past 4 weeks. If answers on ≥ 5 of the items were missing, the total score was set to missing. The available amount and percentage of person-measurement observations was 1197 (78%). Total WRFQ-scores ranged from 0 to 100, with higher scores indicating better work functioning.

### Health status

Fatigue, depressive symptoms, and cognitive symptoms were measured at baseline, 6, 12, and 18 months after RTW. Fatigue was measured with the Checklist for Individual Strength (CIS-8) ‘fatigue severity’ subscale (8 items, α = 0.88) [10]. If answers on > 2 of the items were missing, the total score was set to missing. Total scores were calculated by summing the scores on each item and ranged from 8 to 56, with higher scores indicating more severe fatigue. A score of > 35 was indicative of severe fatigue. Depressive symptoms were measured with the Patient Health Questionnaire-9 (PHQ-9; 9 items, α = 0.88) [11, 12]. If answers on > 3 of the items were missing, the total score was set to missing. Total scores were calculated by summing the scores on each item and total scores ranged from 0 to 27, with higher scores indicating more depressive symptoms. A score of ≥ 10 was indicative of clinical depression. Work-specific cognitive symptoms were measured with the Cognitive Symptom Checklist-Work Dutch Version (CSC-W DV; 19 items, α = 0.95) [13]. This reliable and valid questionnaire [13] originated from the CSC-W21 [14], developed as a self-report measure of cognitive symptoms in the context of work. CSC-W scores are related to work productivity [15] and work functioning [13]. If answers on > 3 of the items were missing, the total score was set to missing. Total scores were calculated by summing the scores on each item, divided by the number of items and then multiplying by 25. Total scores ranged from 0 to 100, with higher scores indicating more work-specific cognitive symptoms.

### Work-related factors

Perceived psychosocial work characteristics were measured at baseline, 6, 12, and 18 months after RTW by the short version of the Copenhagen Psychosocial Questionnaire (COPSOQ) [16] measuring quantitative demands (two items), work pace (two items), influence at work (two items), meaning of work (two items), social support from the supervisor (two items), and social support from colleagues (two items). Total scores were calculated by summing the items for each psychosocial work characteristic and ranged from 0 to 8. Higher values representing higher levels of the measured psychosocial work characteristic. Working hours (per week) were assessed at baseline, 6, 12, and 18 months after RTW.

### Statistical analyses

First, the cancer patients’ socio-demographics, health status, and work-related factors (i.e., mean, median, or percentage) were described. Second, the course of work functioning, health status, and work-related factors during 18-month follow-up was analyzed. Linear mixed models were used to calculate estimated means with corresponding 95% confidence intervals (CIs). Differences between baseline, 6, 12, and 18 months after RTW were tested with pairwise comparisons. Bonferroni correction was used to correct for multiple testing. Third, linear regression analyses with generalized estimating equations (GEE) were performed to examine the associations between health status and work-related factors and the course of work functioning during 18-month follow-up. With GEE, the relationships between the variables of the model at different time-points were analyzed simultaneously [17]. GEE takes the intra-individual correlations between observations into account [17]. An exchangeable structure was found most appropriate after examining the correlation structure of the outcome (WRFQ 2.0) and by comparing the quasi-likelihood under the independence model criterion (QIC), with lower values indicating better fit. Regression coefficients with corresponding CIs for work functioning were presented, which can be used to draw regression lines for cancer patients.

A total of six models were fitted: (1) an unconditional growth model, which included work functioning and a categorical time variable; (2) age, gender, education, and marital status were added to the first model, to control for socio-demographics; (3) health status and (4) work-related factors were added to the second model, respectively; (5) health status and work-related factors were included simultaneously; 6) interaction terms for health status and time and for work-related factors and time were added to the fifth model, one at a time, whereby significant interaction terms (*p* < 0.05) were retained in the subsequent and final model. Data were not imputed; the available amount and percentage of person-measurement observations for work functioning were reported for models 1 and 6. Analyses were performed with SPSS Statistics 23.

## Results

### Characteristics of cancer patients

A total of 384 cancer patients were included in the WOLICA study. At baseline, *n* = 319 (83%) had complete WRFQ, health status, and work-related factors data, *n* = 290 (76%) at 6 months, *n* = 262 (68%) at 12 months, and *n* = 258 (67%) at 18 months after RTW. Cancer patients had a mean age of 50.7 (*SD* = 8.6) years and 63% were female (Table 1). Breast cancer was most prevalent (46%), followed by gastrointestinal cancer (15%), hematological cancer (11%), and urogenital cancer (11%). Seventy-one percent of the cancer patients were treated with systemic therapy exclusively or in combination with radiotherapy and/or surgery. The time between diagnosis and return to work was on average 7 months. Two-thirds of the cancer patients had completed their treatment at baseline.

**Table 1:** Baseline socio-demographics (*n* = 384)

Age in years, *M* (*SD*)	50.7 (8.6)
Gender (female), *n* (%)	243 (63)
Education, *n* (%)	
Low	105 (27)
Medium	129 (34)
High	149 (39)
Marital status, *n* (%)	
Married/cohabitating	305 (80)
Single/divorced/separated	78 (20)
Cancer site, *n* (%)	
Breast cancer	178 (46)
Gastrointestinal cancer	58 (15)
Gynecological cancer	12 (3)
Hematological cancer	42 (11)
Skin cancer	16 (4)
Head and neck cancer	15 (4)
Urogenital cancer	41 (11)
Lung cancer	13 (3)
Other cancer	8 (2)
Type of treatment*, n* (%)	
Surgery	59 (15)
Radiotherapy exclusively, or in combination with surgery	48 (13)
Systemic therapy exclusively, or in combination with radiotherapy and/or surgery	271 (71)
Treatment completed (yes), *n* (%)	246 (64)

At baseline, cancer patients had a WRFQ score of 78.4 (CI: 76.6, 80.2), indicating that on average 22% of the time they had difficulties meeting the demands of the job due to (physical or emotional) health problems (Table 2). Cancer patients worked on average 19.0 (CI: 17.8, 20.1; interquartile range (IQR): 12–24) hours per week at baseline. At that time, they reported a mean fatigue score of 30.2 (CI: 29.0, 31.4; IQR: 22.0–37.8), a mean depressive symptoms score of 4.6 (CI: 4.3, 5.0; IQR: 2.0–7.0), and a mean cognitive symptom score of 24.7 (CI: 23.0, 26.4; IQR: 13.2–24.0).

**Table 2:** Work functioning, health status, and work-related factors at baseline, 6-, 12-, and 18-month follow-up of 384 cancer patients (estimated means with CI)

	Baseline	6 months after RTW	Δ_0–6_	12 months after RTW	Δ_0–12_	Δ_6–12_	18 months after RTW	Δ_0–18_	Δ_6–18_	Δ_12–18_
Work functioning	78.4 (76.6, 80.2)	82.1 (80.2, 83.9)	3.6***	85.2 (83.3, 87.1)	6.8***	3.2**	85.2 (83.3, 87.2)	6.8***	3.2**	0
Health status										
Fatigue	30.2 (29.0, 31.4)	28.2 (27.0, 29.4)	− 2.0**	27.9 (26.7, 29.1)	− 2.3**	− 0.3	27.8 (26.5, 29.0)	− 2.4**	− 0.4	− 0.1
Depressive symptoms	4.6 (4.3, 5.0)	4.3 (3.9, 4.7)	− 0.4	3.8 (3.4, 4.2)	− 0.8***	− 0.5	4.1 (3.7, 4.5)	− 0.6*	− 0.2	0.3
Cognitive symptoms	24.7 (23.0, 26.4)	24.5 (22.7, 26.3)	− 0.23	24.4 (22.7, 26.2)	− 0.3	− 0.1	24.2 (22.4, 26.1)	− 0.5	− 0.2	− 0.3
Work-related factors										
Working hours (p/w)	19.0 (17.8, 20.1)	26.9 (25.8, 28.1)	8.0***	27.2 (26.0, 28.4)	8.2***	0.3	26.2 (24.9, 27.4)	7.2***	− 0.8	− 1.0
Quantitative demands	2.7 (2.5, 2.8)	2.7 (2.5, 2.8)	0.0	2.6 (2.4, 2.8)	0.1	− 0.1	2.6 (2.4, 2.8)	− 0.1	− 0.1	0.0
Work pace	4.5 (4.3, 4.6)	4.6 (4.4, 4.8)	0.1	4.7 (4.5, 4.9)	0.2	0.1	4.6 (4.4, 4.8)	0.1	0.0	− 0.1
Influence at work	4.5 (4.3, 4.7)	4.5 (4.3, 4.7)	0.0	4.4 (4.2, 4.6)	0.1	− 0.1	4.3 (4.1, 4.5)	− 0.2	− 0.1	− 0.1
Meaning of work	6.2 (6.0, 6.3)	5.9 (5.8, 6.1)	− 0.2	5.8 (5.7, 6.0)	− 0.3**	− 0.1	5.8 (5.6, 5.9)	− 0.4***	− 0.2	− 0.1
Social support supervisor	5.3 (5.1, 5.5)	4.8 (4.6, 5.1)	− 0.4***	4.8 (4.6, 5.0)	− 0.5***	0.0	4.7 (4.4, 4.9)	− 0.6***	− 0.2	− 0.2
Social support colleagues	5.6 (5.4, 5.8)	5.2 (5.0, 5.4)	− 0.4**	5.2 (5.0, 5.4)	− 0.4**	0.0	5.2 (4.9, 5.4)	− 0.5***	0.0	− 0.1

*p < 0.05, **p < 0.01, ***p < 0.001. Bonferroni correction was used to correct for multiple testing

### The course of work functioning, health status, and work-related factors

In the first 12 months after RTW, cancer patients reported an increase in work functioning (Δ_0–12_: 6.8) and a decrease in fatigue (Δ_0–12_: − 2.3) and depressive symptoms (Δ_0–12_: − 0.8). Work functioning, fatigue, and depressive symptoms remained stable between 12 and 18 months after RTW. Cognitive symptoms were stable during the first 18 months after RTW.

Cancer patients reported an increase in working hours in the first 6 months after RTW (Δ_0–6_: 8.0). During that period, they reported a decrease in social support from both the supervisor (Δ_0–6_: − 0.4) and colleagues (Δ_0–6_: − 0.4). Working hours and social support from supervisor and colleagues were stable between 6 and 18 months after RTW. Additionally, cancer patients reported a decreased meaning of work in the first 12 months (_Δ0–12_: − 0.3), which was stable between 12 and 18 months after RTW. Quantitative demands, work pace, and influence at work remained stable during the first 18 months after RTW.

### The association among health status and work-related factors and the course of work functioning

The unconditional growth model showed an increase in work functioning in the first 12 months after RTW, and work functioning remained stable between 12 and 18 months (Table 3, model 1; *n* = 1186 person-measurement observations, 77%). Age, gender, education, and marital status did not change the course of work functioning (model 2).

**Table 3:** Association among health status and work-related factors with work functioning over time of 384 cancer patients

	Model 1	Model 2	Model 3	Model 4	Model 5	Model 6
Intercept	78.42 (76.63, 80.22)***	80.28 (71.00, 89.56)***	101.06 (95.26, 106.87)***	68.48 (57.45, 79.51)***	91.97 (83.91, 100.03)***	93.01 (84.58, 101.44)***
Time baseline	Ref	Ref	Ref	Ref	Ref	Ref
6 months	3.62 (1.79, 5.46)***	3.60 (1.73, 5.46)***	2.67 (0.98, 4.35)**	0.88 (− 1.31, 3.08)	1.30 (− 0.61, 3.21)	2.51 (− 0.43, 5.45)
12 months	6.83 (5.09–8.57)***	6.81 (5.05, 8.57)***	5.19 (3.65, 6.73)***	4.03 (2.07, 5.99)***	3.83 (2.06, 5.60)***	0.64 (− 2.03, 3.30)
18 months	6.82 (4.84, 8.80)***	6.74 (4.74, 8.73)***	5.28 (3.58, 6.98)***	4.13 (1.87, 6.38)***	3.98 (2.03, 5.93)***	2.81 (− 0.22, 5.74)
Gender						
Female		0.09 (− 3.16, 3.34)	0.95 (− 1.17, 3.08)	2.31 (− 0.62, 5.26)	2.16 (− 0.17, 4.48)	2.09 (− 0.24, 4.43)
Male		Ref	Ref	Ref	Ref	Ref
Age		− 0.03 (− 0.21, 0.15)	− 0.04 (− 0.15, 0.08)	0.01 (− 0.14, 0.16)	0.00 (− 0.10, 0.10)	− 0.01 (− 0.11, 0.10)
Education						
Low		2.03 (− 1.72, 5.78)	2.47 (0.07, 4.88)*	1.33 (− 1.95, 4.61)	2.42 (− 0.01, 4.85)	2.39 (− 0.03, 4.80)
Medium		− 2.03 (− 5.59, 1.52)	− 0.84 (− 3.23, 4, 1.55)	− 1.75 (− 4.93, 1.44)	− 0.48 (− 2.82, 1.87)	− 0.55 (− 2.88, 1.78)
High		Ref	Ref	Ref	Ref	Ref
Marital status						
Single/divorced		− 1.32 (− 4.85, 2.22)	1.28 (− 1.15, 3.71)	− 1.06 (− 4.13, 2.01)	0.96 (− 1.39, 3.31)	0.98 (− 1.36, 3.31)
Married/cohabiting		Ref	Ref	Ref	Ref	Ref
Fatigue^a^			− 0.19 (− 0.27, − 0.11)***		− 0.16 (− 0.24, − 0.08)***	− 0.16 (− 0.24, − 0.08)***
Depressive symptoms^b^			− 1.34 (− 1.67, − 1.00)***		− 1.15 (− 1.48, − 0.81)***	− 1.13 (− 1.74, − 0.77)***
Depressive symptoms * baseline						Ref
Depressive symptoms * 6 months						0.05 (− 0.56, 0.66)
Depressive symptoms * 12 months						0.35 (− 0.19, 0.90)
Depressive symptoms * 18 months						0.18 (− 0.41, 0.77)
Cognitive symptoms^c^			− 0.41 (− 0.49, − 0.34)***		− 0.39 (− 0.46, − 0.33)***	− 0.40 (− 0.50, − 0.31) ***
Cognitive symptoms^c^ * baseline						Ref
Cognitive symptoms^c^ * 6 months						− 0.06 (− 0.18, 0.06)
Cognitive symptoms^c^ * 12 months						0.08 (− 0.04, 0.19)
Cognitive symptoms^c^ * 18 months						0.02 (− 0.12, 0.16)
Working hours^d^				0.29 (0.17, 0.41)***	0.15 (0.05, 0.25)**	0.14 (0.04, 0.24)**
Quantitative demands^e^				− 2.65 (− 3.33, − 1.97)***	− 0.99 (− 1.54, − 0.44)***	− 0.98 (− 1.53, − 0.42)**
Work pace^e^				− 0.32 (− 0.90, 0.27)	− 0.28 (− 0.76, 0.19)	− 0.26 (− 0.72, 0.21)
Influence at work^e^				0.49 (− 0.03, 1.00)	0.41 (0.00, 0.81)	0.39 (− 0.02, 0.80)
Meaning of work^e^				1.08 (0.36, 1.80)**	0.35 (− 0.25, 0.94)	0.36 (− 0.24, 0.95)
Social support supervisor^e^				1.00 (0.46, 1.55)***	0.64 (0.21, 1.07)**	0.71 (0.29, 1.13)**
Social support colleagues^e^				− 0.45 (− 0.96, 0.06)	− 0.32 (− 0.74, 0.11)	− 0.37 (− 0.78, 0.04)

*p < 0.05, **p < 0.01, ***p < 0.001. Intercept, slopes, and 95% confidence intervals were presented

^a^Range 8–56

^b^Range 0–28

^c^Range 0–100

^d^Per week

^e^Range 0–8

After adding health status (model 3), an increase in fatigue (regression coefficient b: − 0.19; CI: − 0.27, − 0.11), depressive symptoms (− 1.34; − 1.67, − 1.00), and cognitive symptoms (− 0.41; − 0.49, − 0.34) was associated with a decrease in work functioning. When adding work-related factors (model 4), an increase in working hours (0.29; 0.17, 0.41), meaning of work (1.08; 0.36, 1.80), and supervisor social support (1.00; 0.46, 1.55) was associated with an increase in work functioning, while an increase in quantitative demands (− 2.65; − 3.33, − 1.97) was associated with a decrease in work functioning. Changes in work pace, influence at work, and social support from colleagues did not affect the course of work functioning over time. After adding both health status and work-related factors (model 5), the associations remained similar, except for meaning of work; this was no longer associated with the course of work functioning over time.

When including interaction terms between health and work-related factors with time (model 6, *n* = 1106 person-measurement observations, 72%), the interaction terms between cognitive symptoms and time 3 (i.e., 12 months) and depressive symptoms and time 3 were statistically significant. When adding both interaction terms together to the subsequent and final model, both interaction terms remained not significant.

## Discussion

Cancer patients showed an increase in work functioning and a decrease in fatigue and depressive symptoms during the first 12 months and stability between 12 and 18 months after RTW. Cognitive symptoms were stable during the first 18 months after RTW. Working hours increased and social support from supervisor and colleagues decreased in the first 6 months and were stable between 6 and 18 months after RTW. Fatigue, depressive symptoms, and cognitive symptoms were negatively associated with work functioning over time, and working hours and supervisor social support were positively associated with work functioning over time. The effects were the same over time for all variables.

When returned to work, cancer patients experienced difficulties in meeting their job demands due to (physical or emotional) health problems for 22% of their time at work. During the first 12 months after RTW, the amount of time experiencing difficulties decreased to 15% and remained stable between 12 and 18 months. Due to this reduction in experienced difficulties, cancer patients’ level of work functioning 1 year after RTW is similar to the level of work functioning in the general working population [8].

Fatigue and depressive symptoms were decreasing during the first 12 months after RTW and were stable between 12 and 18 months. Even though cancer patients were already below clinical cut-offs for fatigue and depressive symptoms [10, 12], their level of fatigue and depressive symptoms decreased after RTW. Work-specific cognitive symptoms were stable during the first 18 months after RTW. Previous research in cancer patients showed that cognitive impairments are typically subtle, with symptoms across various domains of cognition, i.e., working memory, executive function, and processing speed [18–20]. While acute cognitive changes during chemotherapy are common [19, 20], long-term post-treatment cognitive changes only persist in a subgroup (17–34%) of cancer patients [21]. To gain more knowledge about subgroups of cancer patients with different courses of work-specific cognitive symptoms and their determinants, more research is needed.

The current longitudinal study identified negative associations between fatigue, depressive symptoms, and cognitive symptoms with work functioning over time. A recent systematic review on physical and psychosocial problems associated with difficulties at work in cancer patients beyond RTW showed similar associations, although mainly based on cross-sectional research [22]. The negative associations over time can be interpreted in two ways (i.e., both as a between and a within person effect) [17]. First, cancer patients with fewer health problems (i.e., lower fatigue, depressive symptoms scores, and/or cognitive symptoms) had higher work functioning scores compared to cancer patients with more health problems. Second, a decline in health problems within one cancer patient (i.e., an improvement in health) was associated with an increase in work functioning over time, indicating that an improvement in health can be beneficial for work functioning.

Cancer patients reported an increase in working hours during the first 6 months after RTW, but their working hours were stable between 6 and 18 months after RTW even though they did not reach their contracted working hours (data not shown). The positive association found in this study might be explained by an improvement in health, which allows for better scores on work functioning and for a possibility to work more hours per week. Further research is needed to elaborate this in more detail.

In line with previous research [23, 24], workplace social support decreased over time, especially in the first 6 months after RTW. When examining the association between social support and work functioning over time, higher supervisor support was related to better work functioning over time. Therefore, the observed decrease of workplace social support might negatively affect cancer patients’ work functioning over time. Continuous supervisor social support might be important when guiding and accommodating cancer patients at work. The fact that social support was not associated with work functioning in the general working population [25] suggests that workplace social support is more important for work functioning in vulnerable populations, as has previously been shown [26–28].

Several strengths and limitations have to be addressed. A strength of this study is the longitudinal design with repeated measurements of work functioning, health status, and work-related factors at baseline, 6, 12, and 18 months after RTW. Data were available from all four measurement points for the majority of participants. Another strength is the heterogeneous sample of cancer patients with different cancer diagnoses and treatments. In this study, work functioning scores were positively skewed to the right, both at baseline and at follow-up. Therefore, we used GEE analyses instead of mixed models, which allowed for weaker distributional assumptions [29]. The lack of information about cancer patients who were not asked to participate or were asked but not willing to participate is a study limitation. Consequently, it is not possible to state that the study sample is representative of all cancer patients who resumed work after cancer diagnosis and treatment. Furthermore, this study includes no comparison group of healthy workers or workers with other chronic conditions. In future studies, adding a comparison group could provide additional valuable information to interpret our findings. It is also important to note that both the independent and dependent variables were measured with self-reported measures, which might have resulted in an overestimation of the associations due to shared method variance or shared response biases [30].

In the future, interventions to improve work functioning might be successful when reducing fatigue, depressive symptoms, and cognitive symptoms of cancer patients, because a reduction of these symptoms is related to an increase in work functioning over time. For interventions to reduce cognitive symptoms, it is important to take the specific work situation into account, since cancer patients indicate these cognitive symptoms in relation to work. Furthermore, we have to inform employers and (occupational) physicians about the importance of continuing supervisor social support on a regular basis. To improve work functioning, it is important to monitor cancer patients not only in the period directly after RTW but up to 18 months after RTW, allowing for timely interventions when needed.
